# Bile acids and oxo-metabolites as markers of human faecal input in the ancient Pompeii ruins

**DOI:** 10.1038/s41598-021-82831-y

**Published:** 2021-02-11

**Authors:** Emanuele Porru, Enrico Giorgi, Silvia Turroni, Riccardo Helg, Michele Silani, Marco Candela, Jessica Fiori, Aldo Roda

**Affiliations:** 1 Department of Chemistry “G. Ciamician”, University of Bologna, via Selmi 2, 40126 Bologna, Italy; 2 Department of History and Cultures, University of Bologna, 40124 Bologna, Italy; 3 Department of Pharmacy and Biotechnology, University of Bologna, Via Belmeloro 6, 40126 Bologna, Italy; 4 Department of Humanities and Cultural Heritage, University of Campania “Luigi Vanvitelli”, 81055 Santa Maria Capua Vetere (CE), Italy; 5 Interdepartmental Center for Industrial Research-CIRI-MAM, University of Bologna, Bologna, Italy; 6 INBB-Biostructures and Biosystems National Institute, Viale delle Medaglie d’Oro 305, 00136 Rome, Italy

**Keywords:** Analytical chemistry, Archaeology, Sterols

## Abstract

Small organic molecules, lipids, proteins, and DNA fragments can remain stable over centuries. Powerful and sensitive chemical analysis can therefore be used to characterize ancient remains for classical archaeological studies. This bio-ecological dimension of archaeology can contribute knowledge about several aspects of ancient life, including social organization, daily habits, nutrition, and food storage. Faecal remains (i.e. coprolites) are particularly interesting in this regard, with scientists seeking to identify new faecal markers. Here, we report the analysis of faecal samples from modern-day humans and faecal samples from a discharge pit on the site of the ruins of ancient Pompeii. We propose that bile acids and their gut microbiota oxo-metabolites are the most specific steroid markers for detecting faecal inputs. This is due to their extreme chemical stability and their exclusive occurrence in vertebrate faeces, compared to other ubiquitous sterols and steroids.

## Introduction

Localizing and characterizing organic residues from human activities is a key step in archaeological investigations^1^. Ancient artefacts can contain markers, such as small organic molecules, lipids, proteins, and DNA fragments, that have been preserved over the centuries. Nowadays, new potent and ultrasensitive analytical methodologies can specifically identify these molecules, even in complex matrices, at submolar concentrations. This allows a more detailed study of the historical bio-ecology of humans^2–4^. Researchers have sought to define the most suitable marker molecules for a given archaeological purpose, based on their relationship to specific aspects of ancient lifestyles, such as social organization, daily habits, nutrition, and food storage^5–7^. Coprolites (i.e. fossilized stool) are particulary interesting in this regard because they are usually abundant on archaeological sites and are easily collectable. Ancient organic molecules preserved in human coprolites can provide strategic knowledge about human biology and evolution, allowing researchers to extrapolate information about host health, microbiome structure, diet, lifestyle, and social habits. However, it is challenging to properly assign a human origin to a coprolite sample^8–10^.

As markers of faecal input, sterols are relatively stable even in samples that are thousands of years old^11^. One example is steranes, which are cyclic compounds derived from steroids or sterols. Steranes have been recovered from ancient sedimentary rocks, tracking the diversification and ecological expansion of eukaryotes^12^. However, sterols occur ubiquitously in plants and animal deposits (e.g. root exudates, micro fauna and faeces) and in soils (being produced by soil microorganisms). This ubiquity drastically reduces their specificity as an indicator of a human origin. Researchers have therefore proposed using more steroid molecules, from sexual steroids to bile acids (BAs)^11^.

BAs are likely the most specific steroid markers for determining the origin of a faecal input. This is because they occur exclusively in vertebrate faeces and other biological fluids (e.g. bile and blood). The BA family is a complex and heterogeneous class of compounds, whose qualitative and quantitative composition differs greatly among vertebrates, including mammals. BAs undergo several metabolic reactions by gut microbiota (GM) to produce secondary BAs in the intestine. Oxo-analogues, also called keto-BAs, are steroids with one or more oxo groups in the steroid polycyclic core. Oxo-BAs cover a significant proportion (about 20–30%) of BAs in human faeces and are present only in trace amounts in other fluids such as blood. their qualitative and quantitative composition is mainly related to the GM composition^13^.

Human BAs differ from those of other species, so oxo-BA metabolites can increase specificity in identifying faecal input. BAs and oxo-metabolites are therefore very important for the growing field of bioarcheology due to their stability and specificity (they are present in vertebrate faeces only and are species-specific).

A new mass spectrometry method was recently developed and optimized^14^ for the faecal analysis of BAs, focusing for the first time on oxo-BAs and their metabolic precursors. Here, we modified this method with a new extraction protocol to detect and characterize coprolites in soil samples from the archaeological area of Pompeii. The aim was to confirm the presence of ancient human faecal inputs in samples taken inside the discharge pit of the latrine of the Roman House of M. Obellio Firmo in Pompeii.

This house, placed along the prestigious Via di Nola, was owned by the Obelli family, well known in the city for the public offices held by its members. This ancient building was one of the most important aristocratic residences of Pompeii, provided with two atria and a big garden.

**Figure 1:**
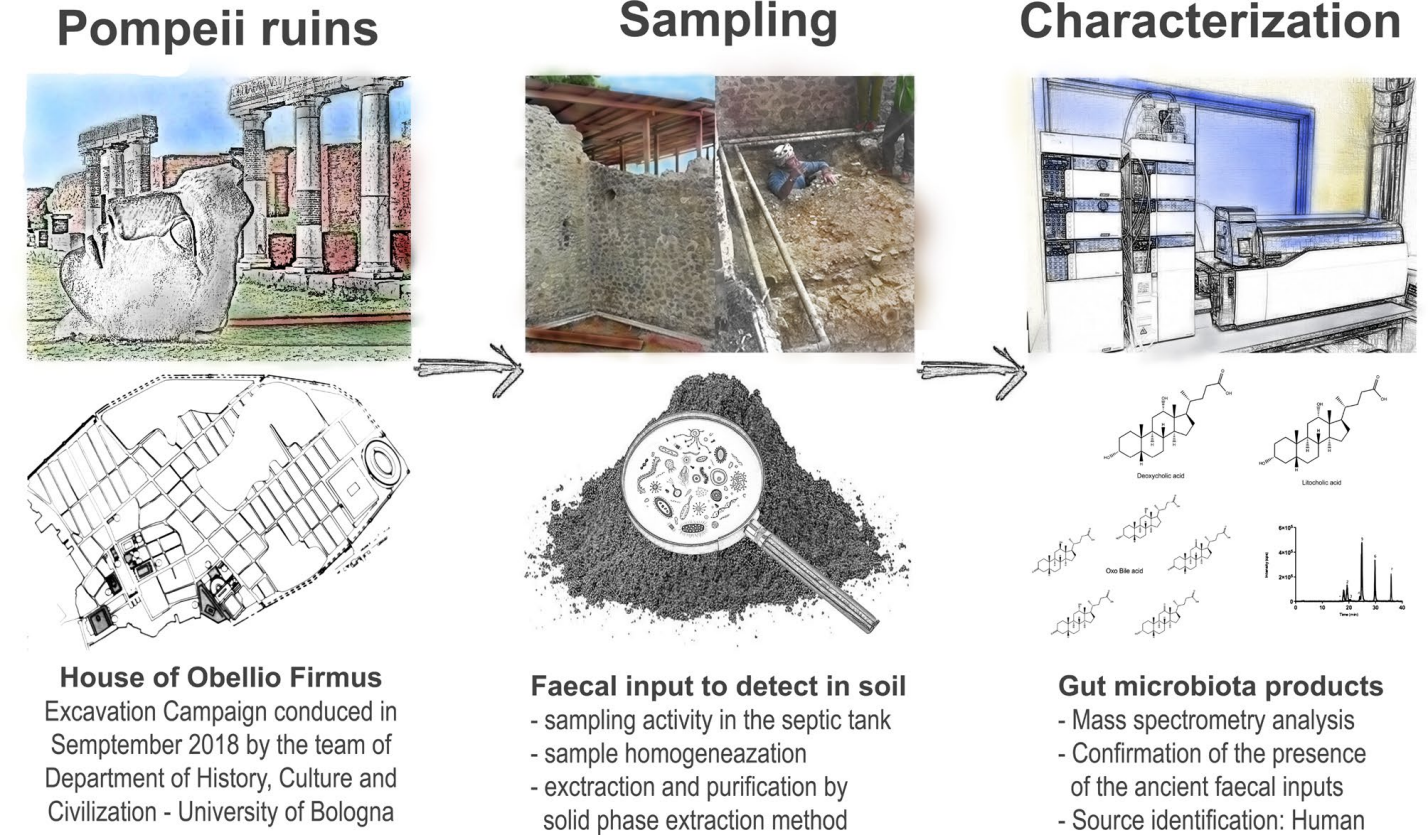
Workflow with steps to characterize the soil samples from Pompeii.

Our aim was to check if the BA and GM metabolite profiles of samples from Pompeii correlated with those of contemporary human faeces. We therefore, analyzed samples from healthy subjects using the same method for BA and oxo-BA metabolites. The literature already reports that steroids, sterols and stanones can be used to identify faecal contamination. However, to our knowledge, this is the first time that a complete BA profile has been used to identify the source of coprolites, faecal input, or similar in archaeology (workflow in Fig. 1), with oxo-BAs of gut microbiota being the most specific markers for faecal detection, since they are present in a relevant amount only in stools.

## Results

The analytical method was based on high-performance liquid chromatography (LC), coupled online to electrospray mass spectrometry (ESI–MS), for the simultaneous determination of 27 BAs, including 21 oxo-BAs that are potentially present in vertebrate faeces and synthesized by gut microbiota enzymes. Isotope dilution was used to quantify the BAs with commercially available deuterium-labelled internal standards of deoxycholic acid (DCA) and lithocholic acid (LCA). Our abbreviations for oxo-BAs reflect the structure of the respective metabolic precursor, indicating the position involved in the oxidative reaction (i.e. ‘3oxo LCA’ means ‘LCA with a hydroxy group converted to a carbonylic group at position 3 of the steroid ring’).

As expected, the studied molecules were extremely diluted in the soil samples from the Pompeii discharge pit. We therefore extracted and concentrated the sample using solid phase extraction (SPE) on C18 silica cartridges to obtain detectable and quantifiable BA levels. Thanks to the extraction procedure, we could detect BAs naturally present in 1 g of faeces even if they were diluted in 1 kg of soil.

Secondary BAs and several oxidized metabolites from the gut microbiota were identified and quantified in the Pompeii discharge pit samples (as reported in Table 1) and in fresh faeces from healthy subjects (n = 10), who were on a regular diet with no history of eating disorders or hepatobiliary diseases (Ethics Committee of St. Orsola Hospital protocol No. 7-2209-USPER).

**Table 1:** BA and BA metabolite content in Pompeii soil and in modern human faeces. The percentages refer to the total content of BAs determined by the LC mass spectrometry.

Compound	Pompeii soil		Human faeces		Pompeii soil	Human faeces
	[pmol/g]	RSD%	[nmol/g]	RSD%	%	%
3,12-Dioxo-cholan-24-oic acid (3,12-dioxo DCA)	1.3	0.7	16.5	0.4	3.7	6.3
12β-Hydroxy-3-oxo-cholan-24-oic acid (12β,3-oxo DCA)	0.5	0.4	3.1	0.3	1.3	1.2
3α-Hydroxy-12-oxo-cholan-24-oic acid (12oxo DCA)	5.1	0.3	41.9	0.5	14.4	16.1
12α-Hydroxy-3-oxo-cholan-24-oic acid (3oxo DCA)	1.1	0.7	13.6	0.4	3.0	5.2
3-Oxo-cholan-24-oic acid (3oxo LCA)	1.5	0.3	15.8	0.4	4.1	5.8
3α,12α-Dihydroxy-7-oxo-cholan-24-oic acid (7oxo CA)	–	–	0.3	0.5	–	0.1
7β-Hydroxy-3-oxo-cholan-24-oic acid (3oxo UDCA)	–	–	0.9	0.4	–	0.4
3α,12α-Dihydroxy-cholan-24-oic acid (DCA)	13.3	0.3	119	0.5	51.9	46.3
3α-Hydroxy-cholan-24-oic acid (LCA)	3.2	0.5	27.5	0.4	15.9	10.2
3β-Hydroxy-cholan-24-oic acid (iso LCA)	1.1	0.8	23.5	0.5	5.8	8.7

As expected, we did not detect the entire spectrum of BAs and derivatives in Pompeii discharge pit (i.e. BAs that are usually less abundant in humans were not detected). Moreover, the absolute content was lower in the ancient sample (soil) than in fresh human faeces, suggesting that the ancient stools were widely dispersed in soil.

However, our results confirmed the presence of mammalian faeces in the ancient samples, whose specific profile of BAs and derivatives is comparable to that of human stools. DCA was the main BA found in the Pompeii discharge pit samples, together with its three derivatives oxidized by the gut microbiota. LCA was detected in high concentration, as was the metabolite 3oxo LCA. Oxo-BAs made up a significant proportion of the detected molecules, up to 25–30% of the total BAs. The slight differences in the BA profiles of Pompeii discharge pit samples and fresh contemporary human faeces can be ascribed to variability in daily BA excretion, related to nutrition, intestinal transit time, and individual gut microbiota profiles. These factors usually slightly modify the faecal BA pool in terms of quantitative content and, to a lesser extent, qualitative composition.

**Figure 2:**
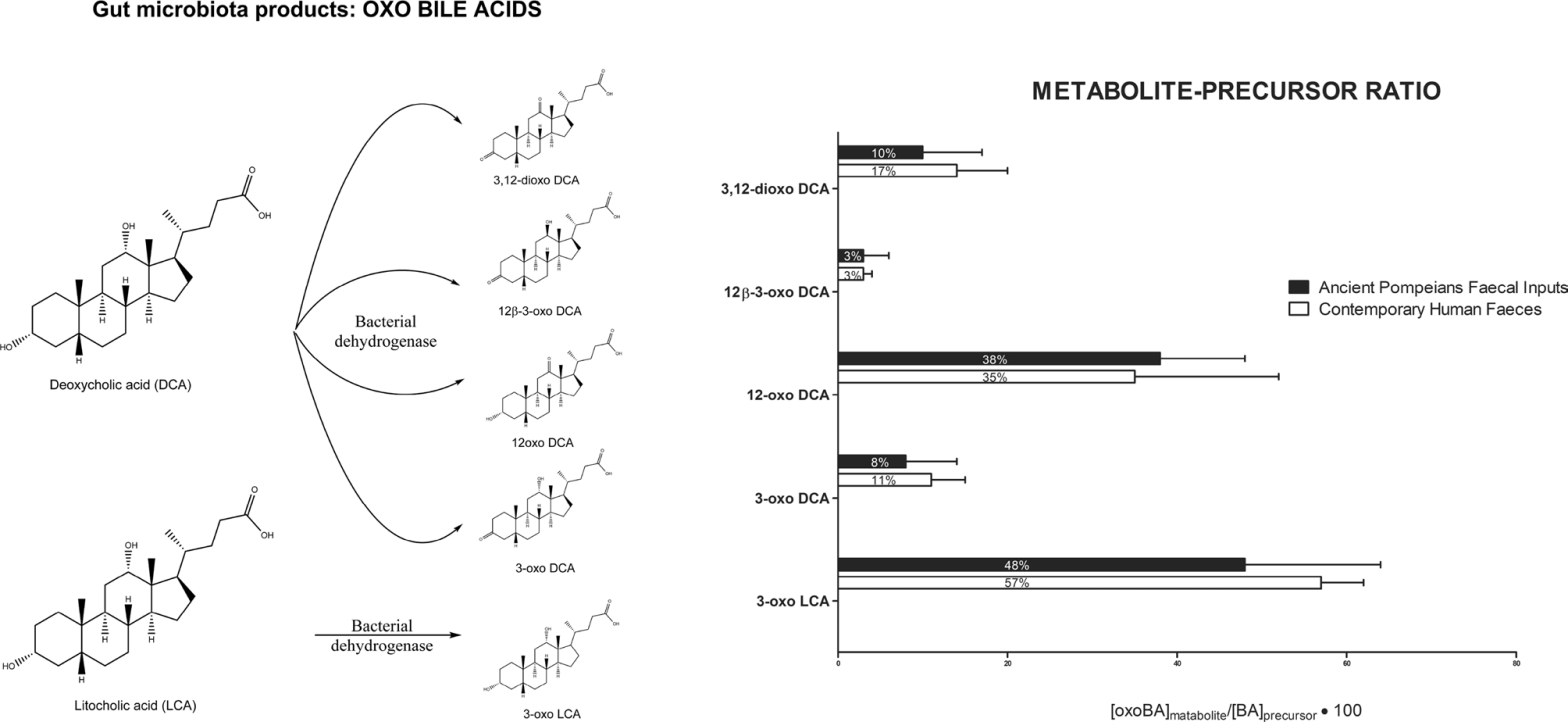
Comparison between the BA profiles in the contemporary human faeces and the ancient faecal inputs collected in Pompeii, using the ratio of the concentration of each metabolite ([oxo-BA] metabolite) to the concentration of its respective metabolic precursor ([BA] metabolic precursor).

The percent ratio between oxidized species (oxo-BAs) and metabolic precursors (BAs) in Pompeii samples was calculated as:

1 $$ \left( {[{\text{oxo - BA}}]_{{{\text{metabolite}}}} / \, [{\text{BA}}]_{{\text{metabolic precursor}}} \times \, 100} \right) $$

and compared with that of the contemporary human faeces (Fig. 2).

This metabolite-precursor ratio was used to normalize and directly compare the results without considering the dilution of the ancient faecal samples in soil, but taking into account the yields of the oxidative reactions by the bacteria hydrogenases in ancient Pompeiians and contemporary subjects.

As shown in Fig. 2, the Pompeii discharge pit sample and contemporary faeces from healthy humans had similar percent ratios of all oxidized metabolites (3,12-dioxo DCA, 12β-3-oxo DCA, 12oxo DCA, 3 oxo DCA, 3 oxo LCA).

No significant differences were found in the quantitative composition (Student's *t* test, P NS, α = 0.05), with a greater variability associated with 3oxo-LCA.

As reported in Table 1, there is greater variability in the data for the qualitative composition of the common BAs, DCA, LCA, and isoLCA that for oxo-BA. This is probably due to the lower stability of these BAs compared to the end-product oxo-BA.

## Discussion

The detection of markers and specific profiles indicating the presence of humans are complementary tools of archaeology, allowing researchers to establish the presence of humans and a given activity conducted at that time.

Markers for coprolites should ideally be able to determine the source of the sample, confirming the ancient presence of humans or animals on a site. However, the chemical composition and stability of faeces over time are still an issue for many endogenous molecules. Predictive and accurate archaeological research must consider the long-term stability of excreted compounds in variable conditions. Matrix composition, temperature, anaerobic oxygen exposure, and many other aspects can affect the chemical profile of ancient samples over the centuries. Neutral steroids such as Δ5-sterols, stanols, and stanones have been widely used to characterize faecal excretion matrix^11^. For example, the different percentages of steroid molecules have been proposed as a way to identify the source of faecal input in soils and sediments^15^. However, sterols occur ubiquitously in soil, which drastically reduces their specificity for this kind of investigation. Furthermore, the complete chemical analysis of steroid compounds is difficult and time-consuming.

**Figure 3:**
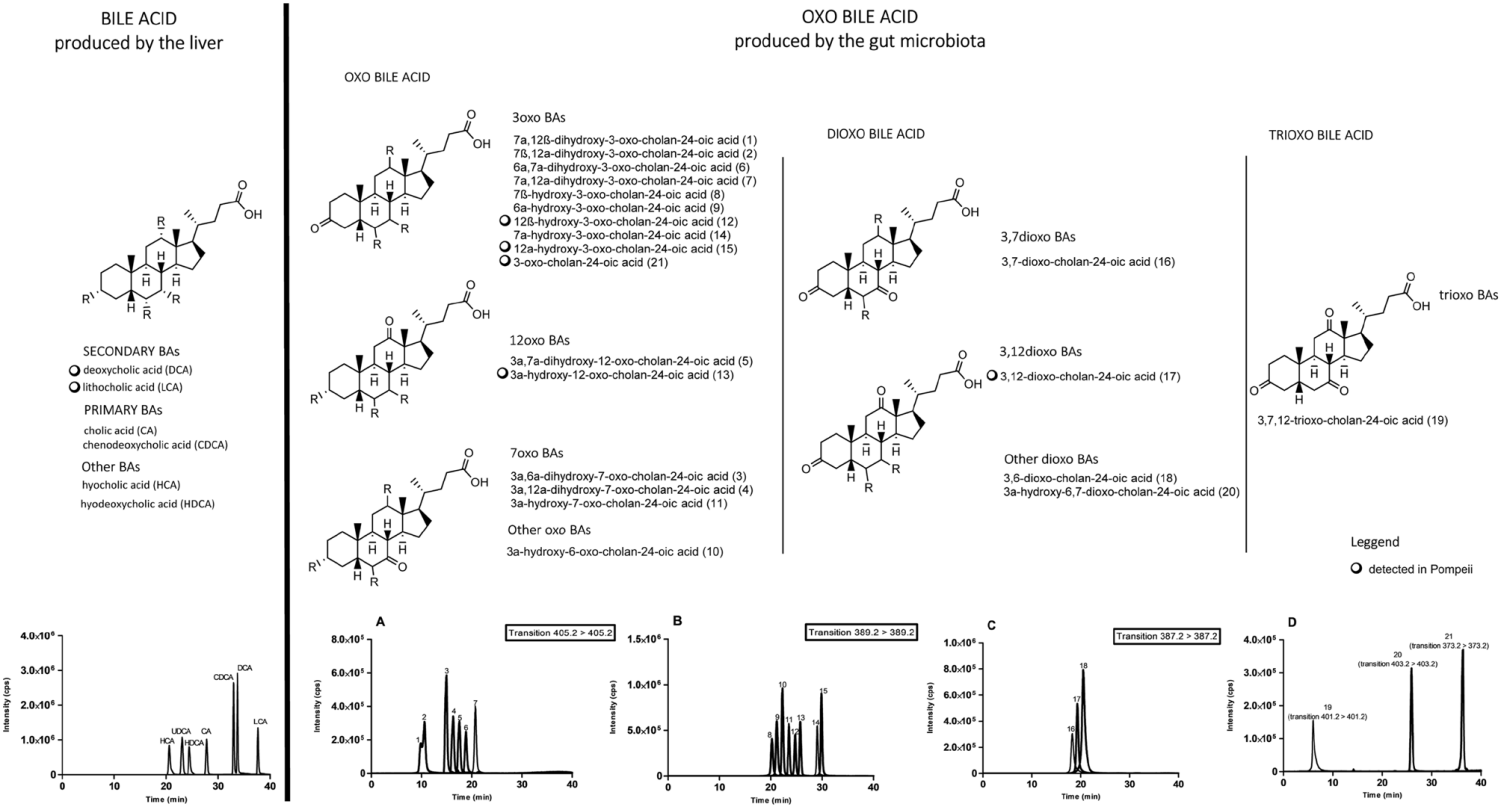
The bile acids and oxo bile acids investigated in the soil samples from Pompeii with chromatograms obtained by LC–ESI–MS. The general structure of the microbiota products from dehydrogenase enzymes are reported subdivided into mono, di e try oxo-metabolites.

BAs are classified as “acidic steroids” and are the end-products of cholesterol catabolism. BAs are absent in plants and invertebrates, which makes them more appropriate than stanols and stanones for characterizing the origin of faecal input. Up to 30–50 different BAs are present in excreted faeces of vertebrates. These BAs differ in three aspects: side-chain structure, stereochemistry of the A/B ring bond, and number and distribution of hydroxyl groups in the steroid nucleus. Structural differences among BAs are also strictly related to the gut microbiota composition^16^. Indeed, BAs undergo several metabolic reactions by the GM, resulting in the production of secondary BAs in the intestine. These reactions include deconjugation, dehydroxylation, oxidation, and epimerization of one or more hydroxyl groups (Fig. 3).

The present study aimed to investigate BAs and oxo-BAs as possible new markers of faecal inputs. To this end, we characterized coprolites that were found dispersed inside the discharge pit of the latrine of the House of M. Obelio Firmo in the archaeological area of Pompeii. Archaeologists identified the latrine’s structure in September 2018 during an excavation campaign in the House garden, in the area behind Room 8 (latrine). Although the building was excavated in the early twentieth century and some external contaminations cannot be excluded (the structure was still connected with the latrine and with other channels in the garden), the pit can be considered a valid context for this study. In 2018, the subterranean structure appeared still unexplored and it can therefore be assumed that the massive organic deposit was the same present at the time of the eruption in 79 A.D. The underground location and the absence of a direct connection with the outside have presumably preserved the deposit from the stress of the heat and the pyroclastic material. A structural failure of the pit vault made it possible to climb down and reach the deep sediment inside. The waste pit’s function is clear. By applying the proposed analytical method, we aimed to profile the sample’s BA composition in pit discharge soils, allowing us to unequivocally identify the presence of human faecal remains. To the best of our knowledge, this is the first time that researchers have profiled the pattern of BAs and derivatives in ancient Romans.

According to our results, a comprehensive characterization of BAs and the complex pool of related metabolites can reliably attribute ancient faecal specimens to human origin. Indeed, the composition of human BAs differs from that of other animal species. Determining the complete pool of BAs and metabolites is thus a powerful tool for differentiating faecal inputs. In this study, we used more specific markers, such as the percent ratio of relevant oxidized species (oxo-BA) and metabolic precursor (BA) concentrations (Fig. 2). This allowed us to disregard the dispersion in soil, and to establish suitable reference values for source identification. They thus allow faeces of human origin to be distinguished from those of other animal species, which have different qualitative and quantitative profiles in terms of BAs and metabolites^17^. This is important because certain animals have coexisted in close contact with humans over the centuries and their remains have been found near human settlements. No less important, BAs are very stable molecules and more resistant to degradation than Δ5-sterols, stanols, and stanones. Thus, BAs can be used where other markers have already been degraded or further metabolized.

BAs have been studied in research fields including medicinal chemistry, environmental chemistry, evolutionary biology, and archaeology. However, this is the first time that ancient coprolites have been characterized for the extraordinary biodiversity of their BAs and BA metabolites in order to determine their likely origin. The composition of BAs and their oxo metabolites is a new approach to increase the specificity of human faecal detection in faecal soil contamination studies. Of the relevant BAs and metabolites, oxo-BA are easily the most stable, therefore our comprehensive and sensitive analytical method for determining these biomarkers is a significant contribution to the growing field of bioarchaeology.

Beyond that, our data indicate a BA profile in human faeces that is well-preserved over the centuries from ancient Rome to the present day. Indeed, our results show that the Pompeian Romans had a BA and BA metabolite profile similar to that of contemporary Italians. Accordingly, we hypothesize that the presence and activity of the microbial enzymes responsible for BA metabolism was similar for these two groups. Interestingly, diet can affect the BA levels in the gut, specifically the levels of chenodeoxycholic acid (CDCA, not found in Pompeii samples) and deoxycholic acid (DCA)^18^. Despite the similarities, we hypothesize that the slight differences in BA profiles, although not statistically significant, may be related to the different amounts of fat in the diet. Changes in diet and nutritional status may slightly but detectably affect these metabolic products as a result of a well-controlled steady-state BA balance in the enterohepatic circulation. Oxo-BAs and other gut metabolites could then be used as a chemical proxy, allowing the structure and functionality of the gut microbiota to be inferred, supporting microbiologists in archaeology and other scientific fields.

## Material and method

### Sampling in the archaeological area of pompei

The latrine was identified in the House of M. Obelio Firmo in Pompeii during the excavation campaign of September 2018. The dimension of the waste pit was 1.80 × 1.10 m, with a sampled volume of soil of 0.84 m^3^. The sampling was conducted at different distances from the expected latrine site to obtain a more representative sample. A total of 10 aliquots of 10 g each were used for BA analysis. The samples were stored at − 20 °C until the analysis.

### Chemicals and solutions

Authentic chemical standards of CA, HCA, CDCA, DCA, DCA 2,2,4,4-d4, HDCA, UDCA,LCA, LCA 2,2,4,4-d4 (all provided as sodium salts) were purchased from Sigma-Aldrich (Saint Louis, USA).

Standards of 3,7,12-trioxo-5α-cholan-24-oicacid, 7 α,12 α-dihydroxy-3-oxo-5 α-cholan-24-oic acid, 3 α,12 α-dihydroxy-7-oxo-5 α-cholan-24-oic acid, 3 α,7 α-dihydroxy-12-oxo-5 α-cholan-24-oic acid, 7 α-hydroxy-3-oxo-5α-cholan-24-oic acid, 3 α-hydroxy-7-oxo-5 α-cholan-24-oic acid, 3,7-dioxo-5α-cholan-24-oic acid, 12α-hydroxy-3-oxo-5α-cholan-24-oic acid, 3α-hydroxy-12-oxo-5α-cholan-24-oicacid, 3,12-dioxo-5 α-cholan-24-oic acid, 3-oxo-5 α-cholan-24-oic acid, 3 α,6 α-dihydroxy-7-oxo-5α-cholan-24-oic acid,3 α-hydroxy-6,7-dioxo-5α-cholan-24-oic acid, 3α-hydroxy-6-oxo-5 α-cholan-24-oic acid, and 3,6-dioxo-5α-cholan-24-oic acid were purchased from Steraloids (Newport, USA).

Standards of 7α,12-dihydroxy-3-oxo-5α-cholan-24-oic acid, 12α-hydroxy-3-oxo-5α-cholan-24-oic acid, 7α-hydroxy-3-oxo-5α-cholan-24-oic acid, 7α,12α-dihydroxy-3-oxo-5α-cholan-24-oic acid, 6α-hydroxy-3-oxo-5α-cholan-24-oic acid, and 6α,7α-dihydroxy-3-oxo-5µ-cholan-24-oic acid were synthesized as reported in the literature^14^.

Stock solutions of each analyte and IS were prepared in isopropanol at a concentration of 1 mg/mL and stored at − 20 °C. These stock solutions were further diluted in isopropanol to obtain working solutions containing all the analytes used for method optimization and calibration curves.

### Sample treatment

Twenty mL of NaOH (0.1 mol/L) and 5 μL of the internal standard solutions (10 mM) were added to each aliquot of 10 g of dried soil samples and incubated for 2 h at 64 °C. The sample was homogenized and centrifuged at 18,000×g for 15 min. The supernatant was collected and extracted using C18 (200 mg, 6 mL) SPE columns. The SPE cartridge was conditioned with 5 mL of methanol and 5 mL of water prior to sample loading. The samples were loaded into the conditioned cartridge, washed with 10 mL of water, and then eluted with 5 mL of isopropanol. The eluate was dried under vacuum, then reconstituted with 100 µL of 40% isopropanol in 15 mM ammonium acetate buffer at pH 8, filtered, transferred to an autosampler vial, and 5 µL injected into the HPLC–ESI–MS system. The recovery of the extraction method was tested with fortified standard soil samples at three concentration levels for each BA.

### Analytical method

BA quantification was carried out with isotope dilution mass spectrometry (ID-HPLC–MS/MS) with deuterium-labelled internal standards, using a method adapted from Franco et al.^14^.

Liquid chromatography was performed using a 2690 Alliance system (Waters, Milford, MA, USA) connected to a triple quadruple mass spectrometer (Quattro-LC, Micromass) with an electrospray interface, operating in the multiple reaction monitoring (MRM) acquisition mode. The performance of the analytical method was tested according to International Conference of Harmonization (ICH) guidelines [Guidance for Industry 2016].

### Statistical analysis

Univariate statistical analysis was performed using GraphPad Prism 5.0 software (La Jolla, CA, USA). A paired *t* test was applied with a significance level of 0.05 (p-value > 0.05 is not significant).
